# Mathematical model for assessing glycemic control in type 2 diabetes mellitus

**DOI:** 10.6026/973206300200116

**Published:** 2024-02-29

**Authors:** Anitha Misquith, Harish Rangareddy, Venkateshappa Chikkanarayanappa, Ayesha Sultana, Srinivasaiah Ashakiran

**Affiliations:** 1 Department of Biochemistry, Sapthagiri Institute of Medical Sciences & Research Center, Bangalore, Karnataka, India; 2 Department of Biochemistry, Haveri Institute of Medical Sciences, Haveri, Karnataka, India; 3 Department of Biochemistry, Sri Madhusudan Sai Institute of Medical Sciences and Research, Chikkaballapur, Karnataka, India; 4 Department of Pathology, St. George's University School of Medicine, St. George's, Grenada

**Keywords:** diabetes mellitus, calculated glycosylated hemoglobin, glycemic control

## Abstract

Glycated hemoglobin (HbA1c) and glycated albumin (GA) are vital markers for assessing glucose control in diabetes. This cross-sectional study involving 901 diagnosed type 2 diabetics aimed to compare calculated HbA1c, using the formula HbA1c = 2.6 + 0.03 x FBS (mg/dL), with directly measured HbA1c. Simultaneously, the study assessed the agreement between the two methods through regression analysis and explored correlations with various measures of glycemic control. The non-parametric Kolmogorov-Smirnov test indicated a non-normal data distribution, prompting appropriate statistical tests. Spearman's correlation coefficient revealed a strong correlation of calculated HbA1c, calculated GA, and estimated average glucose with measured parameters. Wilcoxon rank sum test indicated a significant difference between directly measured and calculated HbA1c (Z -9.487033, p < 0.0001). Passing Bablok regression analysis showed a significant deviation from linearity. Despite the potential cost benefits in resource-poor settings, caution is advised regarding interchangeable use of calculated and directly measured HbA1c in clinical decision-making. Data shows the importance of robust analytical methods in glycemic control assessment, offering insights for managing type 2 diabetes mellitus.

## Background:

Glycated Hemoglobin (HbA1c) is a widely accepted marker for assessing long-term glycemic control in individuals with diabetes [1]. However, calculated HbA1c, determined through specific formula provides an alternative approach that may be more accessible and cost-effective in resource-limited settings [2]. Additionally, glycated albumin (GA) is another marker reflecting short-term glycemic status [3]. Therefore, it is of interest to compare calculated HbA1c with directly measured HbA1c and explore the correlation between calculated HbA1c and various glycemic control parameters in type 2 diabetes mellitus.

## Methodology:

## Study design:

A cross-sectional study involving 901 diagnosed type 2 diabetics was conducted. Universal sampling of patients presenting to the Clinical Chemistry laboratory section of the Sapthagiri Hospital Central Clinical Laboratory for follow-up was done. All individuals diagnosed with type 2 diabetes mellitus (DM) were enrolled in our study, comprising both men and women aged 18 years or older, with hemoglobin levels ranging between 12 and 16 g/dL. This inclusion criterion considered the potential impact of factors such as anemia on HbA1c results. Exclusion criteria encompassed individuals with type 1 diabetes mellitus, hemoglobinopathies, thyroid dysfunction, hypertension managed with diuretics, chronic kidney disease, anemia (hemoglobin <12 g/dL), patients with advanced malignancies and pregnant women. In this study, secondary data analysis was conducted utilizing blood samples collected specifically for fasting blood sugar (FBS), postprandial blood sugar (PPBS), and glycated hemoglobin (HbA1c) assessments. Glucose levels were estimated using the Glucose Oxidase-Peroxidase (GOD-POD) method, a well-established enzymatic assay known for its accuracy and reliability in quantifying blood glucose concentrations [4]. Furthermore, HbA1c levels were measured using the National Glycohemoglobin Standardization Program (NGSP) certified Nephelometry, ensuring adherence to standardized protocols for accurate and consistent results [5]. Mathematical models were employed to derive additional parameters for a comprehensive assessment of glycemic control. Specifically, HbA1c levels were calculated using the formula HbA1c = 2.6 + 0.03 x fasting blood sugar (FBS) in mg/dL [6]. The estimated average glucose (eAG) was concurrently determined using the equation eAG = 28.7 x HbA1c - 46.7, providing a dynamic measure reflecting the average glucose concentration over time [7]. Additionally, glycated albumin (GA) levels were computed using the equation Calculated HbA1c x 2.7, contributing to a multifaceted evaluation of glycemic status [8]. These mathematical models not only facilitate a nuanced understanding of glycemic control but also add depth to the analysis, offering valuable insights into the relationships between different glycemic parameters in the context of type 2 diabetes mellitus.

## Statistical analysis:

Data was tabulated and entered in Microsoft excel. Kolmogorov-Smirnov test indicated a non-normal distribution of data, leading to the application of appropriate non-parametric statistical tests viz., Wilcoxon rank sum test, and Spearman's correlation coefficient and Passing-Bablok regression analysis. Statistical analysis was performed using MedCalc v22.014, and significance was set at p < 0.05.

## Results:

The study cohort had an average age of 54.84 ± 11.57, with a male-to-female ratio of 1.5:1. The glycemic parameters assessed included fasting blood sugar (FBS), postprandial blood sugar (PPBS), and glycated hemoglobin (HbA1c). The mean values for these parameters were as follows: FBS (145.06 ± 65.29 mg/dL), PPBS (212.32 ± 95.84 mg/dL), and HbA1c (7.4 ± 2.08%). Additionally, calculated HbA1c, estimated average glucose (eAG), and calculated glycated albumin (GA) were determined. The calculated HbA1c was found to be 6.95 ± 1.95%, while eAG was 165.79 ± 59.94 mg/dL. The calculated GA percentage was 22.95 ± 6.47%. These findings depicted in Table 1 provide a comprehensive overview of the glycemic control parameters in the study population, offering insights into the potential utility of mathematical models for assessing HbA1c in comparison to directly measured values.|

Spearman's correlation analysis was conducted to investigate the relationships between calculated HbA1c, estimated average glucose (eAG), and calculated glycated albumin (GA) with fasting blood sugar (FBS), postprandial blood sugar (PPBS), and HbA1c levels. The results revealed statistically significant correlations. Calculated HbA1c demonstrated a strong positive correlation with FBS, PPBS and HbA1c. Similarly, eAG exhibited significant positive correlations with FBS, PPBS and HbA1c. Notably, calculated GA also displayed strong positive correlations with FBS, PPBS, and HbA1c as shown in Table 2.

**Figure 1:**
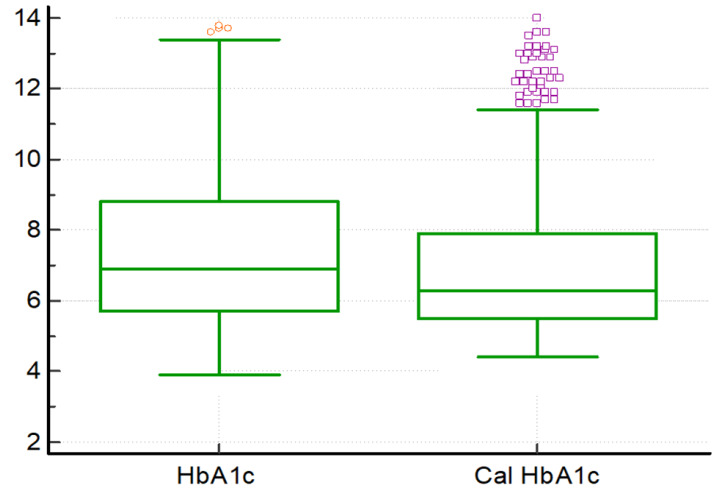
Comparison of the measured HbA1c and Calculated HbA1c using Wilcoxon rank sum test

These findings underscore the consistency and reliability of the calculated parameters (HbA1c, eAG, and GA) in reflecting glycemic status, as evidenced by their strong associations with directly measured FBS, PPBS, and HbA1c levels. The high correlation coefficients and low p-values provide evidence supporting the utility of mathematical models in estimating glycemic parameters, thus emphasizing their potential as valuable tools in clinical practice for individuals with type 2 diabetes mellitus. However, when Wilcoxon rank sum test was employed to compare the levels of HbA1c and calculated HbA1c, revealing distinct patterns in their distribution. The negative ranks (552 instances) indicated that, in a majority of cases, the calculated HbA1c values were lower than the directly measured HbA1c values. Conversely, positive ranks (314 instances) signified scenarios where the calculated HbA1c values exceeded the measured HbA1c values. In addition, there were 35 ties, suggesting instances where the calculated and measured HbA1c values were equivalent. The test statistic Z, calculated as -9.487033, underscored the substantial difference between the inferential method and direct measurement of HbA1c. The obtained p-value, less than 0.01, indicated statistical significance, providing robust evidence for the discrepancy between the two methods as depicted in Figure 1. These findings emphasize the importance of considering the methodological approach in determining HbA1c levels, with potential implications for clinical interpretation and patient management.

**Figure 2:**
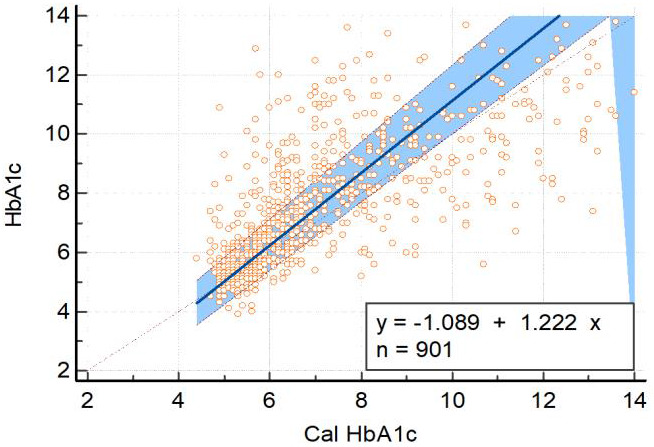
Passing Bablok regression analysis of measured HbA1c and calculated HbA1c

Passing-Bablok regression analysis was conducted to assess the concordance between HbA1c and calculated HbA1c, revealing systematic, proportional, and random differences between the two variables. The regression equation, y = -1.088889 + 1.222222 x, exhibited a systematic intercept difference (A) of -1.0889, with a 95% confidence interval (CI) ranging from -1.5533 to -0.7000. The proportional difference in slope (B) was 1.2222, and the 95% CI ranged from 1.1562 to 1.3043. These systematic and proportional differences indicate a consistent bias between HbA1c and calculated HbA1c as shown in Figure 2.

The residual standard deviation (RSD) was 1.0673, and the 95% CI for the ± 1.96 RSD intervals ranged from -2.0919 to 2.0919, indicating random differences between the observed and calculated values. The Cusum test for linearity revealed a significant deviation from linearity (P<0.01), suggesting potential non-linear associations between HbA1c and calculated HbA1c. These results underscore the presence of systematic, proportional, and random differences, as well as deviations from linearity, between the two measurement methods.

## Discussion:

Our study findings and the study by Musa IR *et al.*, collectively highlight the complexities and challenges associated with estimating HbA1c through mathematical models in individuals with type 2 diabetes mellitus. Musa IR *et al.* reported a borderline difference in mean calculated and measured HbA1c levels, accompanied by a significant correlation but no agreement between the two methods. The Bland Altman plot analysis indicated a bias with limits of agreement, emphasizing the discrepancies in their measurements [2]. Our study, corroborating the high correlation coefficients, acknowledges the potential utility of mathematical models in estimating glycemic parameters. However, the Wilcoxon rank sum test revealed distinct patterns in the distribution of HbA1c and calculated HbA1c levels. The majority of cases exhibited lower calculated HbA1c values, as indicated by negative ranks, while positive ranks suggested instances of higher calculated HbA1c values. Ties, where the values were equivalent, were also observed. The statistically significant test statistic (Z = -9.487033) and p-value (< 0.01) underscore the substantial difference between the two methods. Passing-Bablok regression analysis further elucidated systematic and proportional differences between HbA1c and calculated HbA1c. The unreliability of calculated HbA1c raises concerns, as it may hinder accurate assessments of glycaemic control, particularly in patients with diabetes mellitus (DM). Notably, variations in HbA1c levels, influenced by factors such as age, sex hormones, visceral fat distributions, and socioeconomic status, contribute to the complexity of interpreting glycaemic control [10]. While some studies recommend calculated HbA1c based on self-measured glucose for assessing glycaemic control [11], our results question the interchangeability of calculated and measured values. The observed correlation between the two may be attributed to higher HbA1c levels in patients with persistently elevated blood glucose, especially in uncontrolled DM. However, our study, adopting a commonly used formula, contradicted findings from similar approaches, emphasizing the need for caution. Other equations for estimating HbA1c have been explored, demonstrating significant correlational differences [12]. In the study by Khan HA *et al.*, the following regression equations were employed: HbA1c = 0.387 (FBS) + 4.855 and FBS = 1.33 (HbA1c) - 2.528, for the purpose of inter-converting FBS and HbA1c levels, providing a predictive framework for their anticipated values in individuals with diabetes. Furthermore, the regression equation established in this particular study indicated that the cut-off point of HbA1c (6.5%) corresponds to an FBS level of 6.1 mmol/L. This FBS level is notably lower than the conventional diagnostic cut-off point for FBS, set at 7.0 mmol/L [12]. Interestingly, Colagiuri *et al.* have previously illustrated narrow glycemic threshold ranges associated with diabetes-specific retinopathy. Their findings suggested a potential revision of the existing diagnostic level for FBS to 6.5 mmol/L and thus aligning it with the HbA1c criterion of 6.5%, thus proposing an alternative and comparable diagnostic criterion for diabetes [13]. This observation prompts a reconsideration of diagnostic thresholds and highlights the potential interchangeability of HbA1c and FBS levels in diabetes diagnosis, indicating a need for further exploration and validation of these diagnostic criteria. However, the clinical acceptability, assessed through limits of agreement, was not uniformly addressed. Some studies restricted the use of calculated HbA1c to well-controlled DM cases, acknowledging its limitations in broader applications [6,14]. Desai NG *et al* *et al.* demonstrated that HbA1c values derived from current blood glucose and past HbA1c levels do not precisely match the HbA1c values found in erythrocytes. Consequently, they suggested that this formula is suitable for patients with well-controlled diabetes only and should not be considered a substitute for estimated HbA1c [14]. Various factors, both pathological and physiological, can influence the outcomes of HbA1c and should be taken into account during result interpretation. These factors encompass hemoglobinopathies, uremia, pregnancy, hemodialysis, alcohol consumption, and the administration of aspirin [15]. The decision to use calculated HbA1c may be justified by practical considerations, such as the challenge of frequent HbA1c monitoring, cost issues, and the simplicity of the adopted formula. However, our results question this practice, emphasizing the discrepancy between calculated and measured values. While the measured HbA1c test is relatively expensive, the importance of reliable results cannot be understated. Koga M *et al.* demonstrated that the computation of HbA1c and glycated albumin (GA) values using newly developed formulas based on self-monitoring of blood glucose (SMBG) data exhibited overall consistency with the measured values. These calculation formulas allowed for the estimation of HbA1c and GA values through the systematic analysis of sequentially collected SMBG data [16]. The formula for calculated GA in our study was derived from the study by Yoshiuchi *et al.* which reported that the GA to HbA1c ratio in the patients with type 2 diabetes mellitus was 2.7 [17]. The glycation gap (GGap) refers to persistent discrepancies between glycated hemoglobin and actual glycemia determined from fructosamine or mean blood glucose. This incongruity is observed in a significant percentage of individuals with diabetes, exceeding 1 unit of glycated HbA1c% or 7.2 mmol/mol in nearly 40% of assessments [18]. Consequently, glycated albumin may serve as a more effective predictor of mean blood glucose. In our study we found a significant correlation between calculated GA and measured glycemic parameters. However, the glycated albumin was not measured and the glycemic excursions were not considered. By incorporating these factors into mean blood glucose (MBG), one can anticipate achieving values that more accurately resemble measured glycated albumin (GA) values. Alzahrani N *et al.* determined that a moderate and statistically significant positive correlation exists between fasting blood sugar (FBS) and the estimated average blood glucose (eAG) calculated from HbA1c. While FBS may serve a purpose in the daily monitoring of diabetes, additional investigations are necessary to establish conclusive evidence supporting the potential replacement of HbA1c by FBS and its derived variable eAG as indicators for long-term overall control in patients with Type 2 Diabetes Mellitus (T2DM) [19]. The estimated average glucose (eAG) transforms the HbA1c percentage into an average blood glucose level, measured in the units displayed on glucose meters used for daily self-monitoring (mg/dL), allowing diabetic patients to relate more closely to their monitoring results. Similar to HbA1c, eAG assesses the overall effectiveness of a patient's glucose level management, providing valuable insights for patients to comprehend the long-term progress of their treatment [20]. The most extensive study exploring the relationship and correlation between HbA1c and eAG is credited to the HbA1c-Derived Average Glucose (ADBG) Study Group, which developed the corresponding formula [7,21]. Our study emphasizes a robust positive correlation between eAG and markers of glycemic control, namely FBS, PPBS, and HbA1c. Importantly, patients can now enhance their comprehension of glucose monitoring by drawing connections between HbA1c and eAG results. Given the additional benefits that eAG contributes to patient care, calculated eAG levels is included alongside HbA1c values in laboratory reports.

## Conclusion:

The use of mathematical models for assessing glycemic control in type 2 diabetes mellitus by highlighting the consistency and reliability of the calculated parameters (HbA1c, eAG and GA) in reflecting glycemic control, as evidenced by their strong correlation with directly measured FBS, PPBS and HbA1c levels is shown. While calculated HbA1c and GA may offer cost-effective alternatives, caution is warranted in their interchangeable use for clinical decision-making. The study emphasizes the need for further research and validation to establish the robustness of these mathematical models in diverse clinical settings. Further analyses and validation studies are warranted to explore the clinical implications and accuracy of these calculated values in diverse patient populations.

## Funding:

There was no funding for this study.

## Figures and Tables

**Table 1:** Glycemic control parameters of the study population

**Parameter**	**Mean ± SD**
FBS (mg/dL)	145.06±65.29
PPBS (mg/dL)	212.32±95.84
HbA1c (%)	7.4±2.08
Calculated HbA1c (%)	6.95±1.95
eAG (mg/dL)	165.79±59.94
Calculated GA (%)	22.95±6.47

**Table 2:** Correlation of calculated parameters with measurands of glycemic control

**Spearman rank correlation coefficient**	**FBS**		**PPBS**		**HbA1c**	
	**rho**	**P value**	**rho**	**P value**	**rho**	**P value**
Calculated HbA1c (%)	1	<0.01	0.858	<0.01	0.79	<0.01
eAG (mg/dL)	0.79	<0.01	0.793	<0.01	1	<0.01
Calculated GA (%)	1	<0.01	0.859	<0.01	0.79	<0.01
